# Negative Symptoms of Schizophrenia: Contribution of IL-1β, IL-4, and IL-10 Gene Polymorphisms and Adverse Childhood Experiences

**DOI:** 10.1192/j.eurpsy.2025.2218

**Published:** 2025-08-26

**Authors:** V. Mikhailova, V. Golimbet, M. Alfimova, V. Plakunova, T. Lezheiko

**Affiliations:** 1 Clinical Genetics Laboratory, Mental Health Research Center, Moscow, Russian Federation

## Abstract

**Introduction:**

Schizophrenia is a severe mental illness manifested by various symptoms. Negative symptoms (NS) are associated with disability and poor function of patients. The study of NS neurobiology is complicated by their heterogeneity. Factor analysis revealed two distinct NS subdomains with different pathophysiological mechanisms: volitional pathology, including avolition and apathy (AA), and diminished expression (DE). Inflammation is one mechanism that may underlie NS, including their heterogeneity.

**Objectives:**

To investigate the differentiated associations between polymorphisms of interleukin genes IL-1β (rs16944), IL-4 (rs2243250), and IL-10 (rs1800872, rs1800896) and adverse childhood experiences (ACE) with NS of schizophrenia, specifically the factors of AA and DE. We hypothesize that genetic variants, which may aggravate the inflammatory response, are associated with higher NS and NS factors scores.

**Methods:**

Data from 564 patients diagnosed with schizophrenia or schizoaffective disorder were included in the study. NS factors were calculated based on the Positive and Negative Syndromes Scale. The two-way ANOVA (sex, genotype) with Bonferroni post hoc test was used to examine the effect of the genotypes on the PANSS-derived NS subdomains.

**Results:**

The high-expressive allele of IL-1β and low-expressive alleles of IL-4 and IL-10 are associated with more severe NS. However, a differentiated association with the AA and DE factors was found only for the IL-10 rs1800872 polymorphism. Among carriers of the low-expressive AA allele with ACE, there is a trend towards increased ED scores, but not AA scores.

**Conclusions:**

The findings confirm that the imbalance between pro-inflammatory and anti-inflammatory cytokines may be a key mechanism influencing the severity and heterogeneity of NS.

**Disclosure of Interest:**

None Declared

